# Experiences and lessons learned from a global research administration capacity building project to support and expand HIV/AIDS research in southwestern Uganda

**DOI:** 10.1007/s44250-024-00118-1

**Published:** 2024-07-16

**Authors:** Francis Bajunirwe, Lauren B. Armstrong, Jacqueline Karuhanga, Margaret Mbabazi, Ezrah Muhindo, Amber Steen, Elizabeth W. Olmsted

**Affiliations:** 1 Department of Community Health, Mbarara University of Science and Technology, Mbarara, Uganda.; 2 School of Medicine Office of Grants and Contracts, University of Virginia, Charlottesville, VA, USA.; 3 MUST Grants Office, Mbarara University of Science and Technology, Mbarara, Uganda.; 4 Center for Global Health Equity, University of Virginia, Charlottesville, VA, USA.; 5 Department of Pediatrics, Virginia Commonwealth University, 1008 East Clay Street, B-022, Box 980270, Richmond, VA 23219, USA.

**Keywords:** Research capacity, Global health, International research

## Abstract

International collaborative research projects conducted at academic research institutions, including complex basic science, clinical, and translational HIV/AIDS research, require intensive communication, coordination, and thoughtful relationship-building at all institutional levels and especially require the support of experienced and well-trained Research Administrators. To be successful, global research teams must be not only scientifically adept, but supported by a staff skilled in identifying opportunities, submitting proposals, and managing all aspects of award administration and reporting. Using a National Institutes of Health, Fogarty International Center funded training grant, the project team aimed to develop a comprehensive Research administration capacity-building program that would improve the support provided to investigators at Mbarara University of Science and Technology in Uganda through collaboration with the University of Virginia in the United States and expand future funding success for innovative HIV/AIDS and HIV-related research. The experiences and achievements of conducting a comprehensive, primarily remote training program are documented for other global partnerships to highlight successes and uncover opportunities to ensure sustainability. Despite the hurdles of the COVID-19 pandemic, this program was able to succeed through a long-term relationship of mutual respect between the two institutions. Project success was achieved by the team’s unwavering commitment to collectively explore and implement various methods to achieve its goals.

## 1 Introduction

The incidence of HIV in Uganda has decreased significantly over the past two decades due to concerted efforts in treatment and prevention [1, 2] however, cases remain high in certain subgroups such as adolescent girls and young women [3]. With the availability of effective treatment, persons living with HIV are living longer [4] and facing new health challenges such as non-communicable diseases [5]. Therefore, HIV remains a major public health threat and there is a need to understand the existing challenges of a high incidence and grapple with the emerging challenges of long-term survivors, namely, increased risk for non-communicable diseases such as cardiovascular disease and malignancies among patients receiving antiretroviral therapy.

Although the need to conduct research on HIV remains steady, the administrative infrastructure to support these critical HIV studies continues to be inadequately developed in many countries with high incidence and burden of HIV disease in sub Saharan Africa [6]. Many institutions do not have sufficient capacity and support for pre-award activities such as awareness of grant opportunities, plans for grant writing and submission, budget development, or infrastructure to support collaborative teams that generate new research grants for an institution. Additionally, when an award is received, these institutions may lack sufficient experience in post-award activities such as grants management and reporting.

Mbarara University of Science and Technology (MUST) in southwestern Uganda and the University of Virginia (UVA) in the United States have enjoyed nearly a decade of research collaboration focusing on infectious diseases, particularly tuberculosis and HIV. This collaboration has borne multiple fruitful grant proposals, therefore to ensure a smooth administration of research grants, MUST established a Grants Office (MGO) tasked with presiding over all pre- and post-award activities at the institution. However, like most institutions in resource limited settings, MUST needed capacity building to support research administration.

In 2019, MUST and UVA leveraged the longstanding multidisciplinary collaboration to successfully compete for a research administration capacity-building grant from the US National Institutes of Health’s Fogarty International Center (G11TW010948) with the aim to develop and implement a long-term mentored training program for the MGO. The capacity building plans targeted all aspects of research administration including activities to increase research opportunity awareness, grant writing, and submission support for HIV research at MUST. The aims of the program were designed to ensure an increase in the number of HIV-related proposal submissions and to develop and implement a longitudinal mentored research administration training program at MGO. The intended outcomes of the program included expanding proactive administrative support services, ensuring compliance and accountability, and guaranteeing timely and effective reporting to the relevant stakeholders. The purpose of this paper is to document experiences, achievements, and lessons learned and evaluate the successful implementation of this collaborative capacity building program for HIV research administration at Mbarara University.

## 2 Material and methods

### 2.1 Setting

The research administration capacity building program was conducted at Mbarara University of Science and Technology in rural southwestern Uganda. MUST, established in 1989, is the second largest public university in Uganda and serves a student population focused on interdisciplinary and community-oriented training. Aiming to position itself as a prime recipient of NIH research awards, MUST increased its US-based research revenue from $1.5 M in 2009, when the MGO was formed, to $5.8 M in 2018. To support the administrative needs of the faculty and research community, the MGO leveraged the existing partnership with researchers and administrators at the University of Virginia, a research-intensive academic institution in the United States. Aligning with the goals of the Fogarty International Center’s G 11 program, this collaboration sought to draw on the experiences of both teams to create and implement an administratively focused capacity building and training program.

### 2.2 Design

**Fig. 1:**
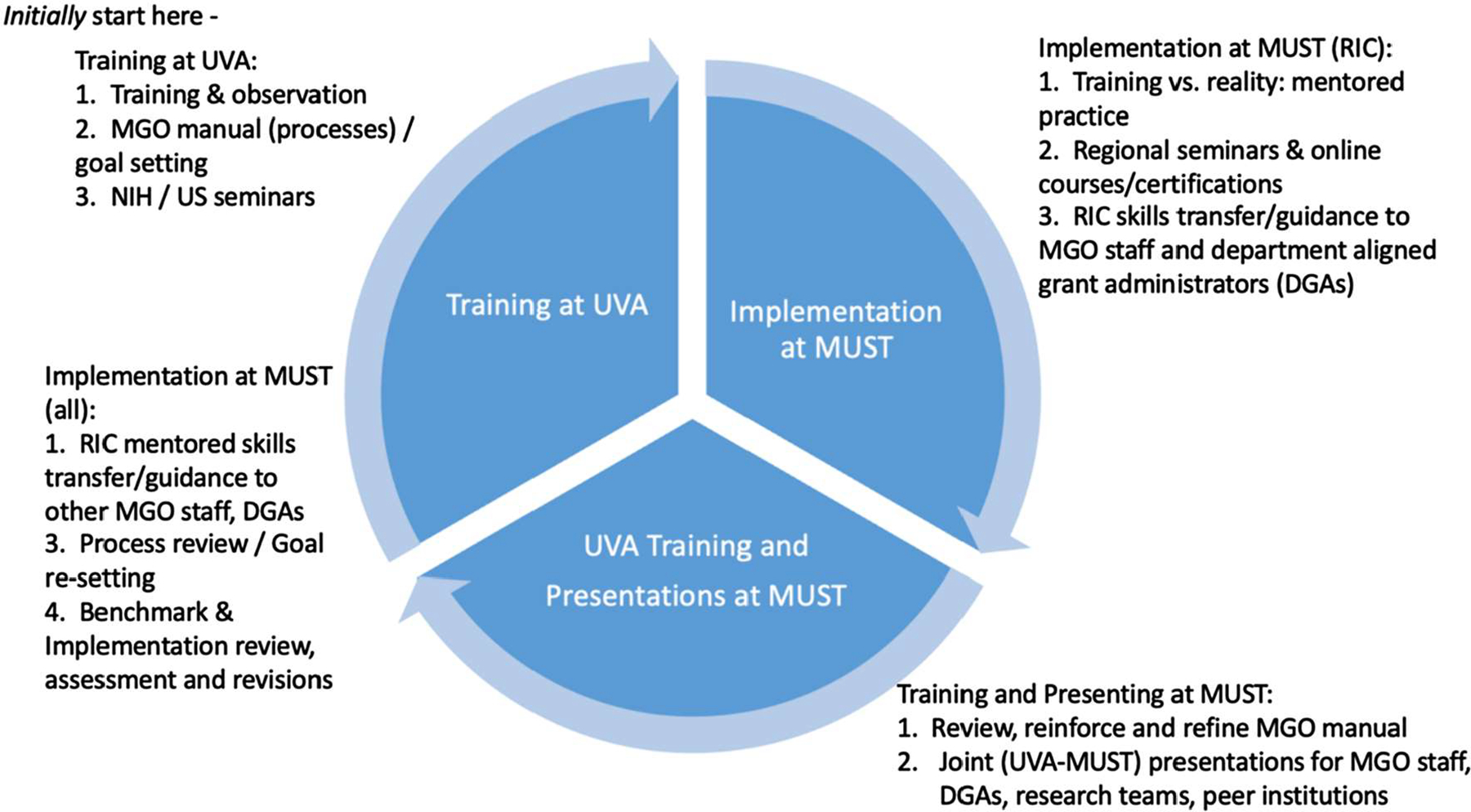
Project cycle with training sequence and methods. Starting in the top left quadrant, this figure shows the various methods and activities employed throughout the 3+ year project

Ethical approval was obtained by the MUST research ethics committee. The proposed methods for implementing the research administration training program included a quantitative needs assessment survey of MUST stakeholders, in-person training, attendance at relevant training conferences, training certifications via online programming, and regularly scheduled video conferencing for virtual training and engagement. Our implementation approach was organized in three phases each year as is shown in Fig. 1.

#### 2.2.1 Project planning and initial training

Initial planning and training activities in the first year of the program were conducted virtually, in advance of the MGO team’s first visit to UVA. The focus of this visit was meeting with the UVA training team, researchers from UVA who have held K awards and those who supported international trainees, and departmental and central office grant administrators. The MUST-UVA team also met with UVA leadership involved in promoting global research collaborations. During this two-day intensive program, the team from MUST participated in presentations, one-on-one meetings, networking events, and training workshops. As a second part of the initial visit, the MUST and UVA teams travelled to a professional conference, attending various subject-based seminars as well as unstructured events to facilitate informal relationship-building within the team. The group also participated in collaborative opportunities for idea sharing and peer-based learning. The final activities of the visit were to update the project work plan and prioritize the training curricula and future events.

#### 2.2.2 Needs assessment survey

To benchmark the current state of research administration at MUST, we disseminated two needs assessment surveys, one to active and potential researchers and one to senior, central, and department level research administrators. The survey targeted to researchers asked eleven questions related to current and pending research proposal activity, barriers to success, and suggestions for how to improve research and research administration support. We received responses from 16 researchers who completed the entire survey. The second needs assessment survey was sent to pre- and post-award research administrators which asked ten questions related to current role, number of proposals supported, time spent on pre-award activities, responsibilities, and suggestions for additional training and support. We received complete survey responses from ten research administrators. Questions within the administrators’ survey were designed to elucidate the experience level of the participants, duties, perceived needs, and suggestions regarding training gaps.

### 2.3 Training, implementation and practice

#### 2.3.1 Training

The COVID-19 pandemic severely impacted the proposed primary method of training by prohibiting travel and in-person activity. To accommodate this disruption, the teams shifted to a fully virtual meeting and training schedule, including twice monthly meetings. The team utilized the shift to virtual interaction to build on the stated aims of the proposed program of a “train the trainer” methodology. A year-long monthly presentation series, which included presentations by the MUST and UVA teams, as well as by members of the Training Advisory Committee (TAC), allowed for simultaneous training from UVA and the MGO team and subsequent training dissemination developed for all MUST researchers, pre- and post-award research administrators, and leadership. The TAC was comprised of six members with representation from MUST, UVA, Moi University in Kenya, and the Joint Clinical Research Centre in Uganda.

#### 2.3.2 Implementation through dissemination and outreach

Key results were disseminated through direct outreach to the MUST research community including all other MGO staff, departmental research administrators, active and aspirational researchers, MUST leadership, and also colleagues at peer institutions within the local region. An enhanced MGO website, in-person presentations, and educational opportunities, including mentoring faculty and staff in pre- and post-award research administration, were the primary outcomes.

#### 2.3.3 Practice—program review with the TAC members

Through scheduled reviews with the Training Advisory Committee, the MUST and UVA program team sought recommendations and suggestions for program improvement, revision, and focus. The TAC members were also invited to participate in program and training activities.

## 3 Results

### 3.1 Project planning and initial training

The MUST team traveled to UVA in October 2019 to kick-off the program and for an initial intensive training session with the UVA project team which included two MPl’s, Co-l’s, and faculty mentors. The agenda included meetings with UVA’s key research administration stakeholders at both the departmental, School, and Central office levels to gain insight into best practices of a highly ranked US public institution.

During this visit, the MGO team met with:

- UVA’s Research Development Office, within the Office of the Vice President for Research, to discuss the process of managing and competing internal grants, grant search engines, and how to create a collaborative researcher-administrator team.
- UVA’s Clinical Translational Science Award leadership team for an in-depth tour of UVA’s NIH-funded, web-based multidisciplinary training portal and K-award development program and the various ways in which MUST could develop similar tools to support its research community.
- UVA’s Office of the Vice Provost for Global Affairs to discuss the importance of international collaborative research and the MUST-UVA relationship.
- UVA Infectious Disease researchers and administrators to review management and reporting requirements for the recently awarded UVA-MUST partnering U01 and building a multi-institution mentoring team and competing for mentored career development awards like the NIH “K” awards.
- UVA’s Office of Sponsored Programs, where discussions focused on UVA’s post-award and subcontracting practices for foreign subawardees. This meeting created a framework of understanding to inform updated practice, documentation, and reconciliation processes. The G 11 team independently continued to review UVA’s financial reporting requirements and documentation, drafting the initial standard procedures for reporting and invoicing on cash-advance-based subcontracts between UVA and MUST.

The MUST and UVA teams then traveled to San Francisco, California to attend an annual professional research administration conference. In-person workshops attended by MUST and UVA team members included: Proposal Development: Pre-Award Overview; Pre-Award Decisions that Impact Post-award; Closing the Gap Between Pre and Post Award Offices; Post Submission to Award Acceptance; Grant Training for Dummies; The Electronic Research Administration (eRA) Landscape: Approaching Strategically; Best Practices for Effective Foundation Proposals; Getting an Award: It is More than Finding a Suitable Funding Opportunity; Designing a Training Program for your Institution: Techniques, Tools and Tips; Creating Vibrant Research Administration Teams, No Matter the Culture, Priorities and Personalities; and Navigating the Organizational Impact of Recent Changes in Federal Regulations and Policy.

On the final day of the conference, the group gathered to update the G 11 work plan, prioritize the training curricula and future activities, and evaluate the information gathered to date.

### 3.2 Needs assessment survey

The responses to the needs assessment survey highlighted many strengths of the MGO’s existing Research administration services, including reliable and efficient post-award management. The respondents also highlighted multiple areas for improvement, including more direct and transparent communication during research development and proposal preparation, streamlined and predictable processes and administrative tools, and better collaboration between the MGO and researcher community to enable more collaborative intra-institutional initiatives. Predictably, both the research administration and researcher populations noted similar needs for more automation and better/earlier process intervention. However, the administrative staff highlighted mentorship and more clearly defined roles and strategic planning as anticipated springboards to success. The researchers focused on research development tasks, such as biostatistical support and pre-award administrative process management, as the most likely to enhance success. We assessed progress through a feedback survey at the end of the training program rating the perceived effectiveness of our training sessions and by tracking grant submissions and awards (see Table 4) which highlights an increase in NIH submissions, the number of HIV/AIDS related awards, and total number of awards over the four year project period.

The researcher respondents were asked about their current grant award and proposal submission activity and responses are shown in Table 1 below. It is noted that most of our respondents were seasoned researchers engaged with the research office. These results indicate a need to reach and engage more with junior and aspirational researchers.

**Fig. 2:**
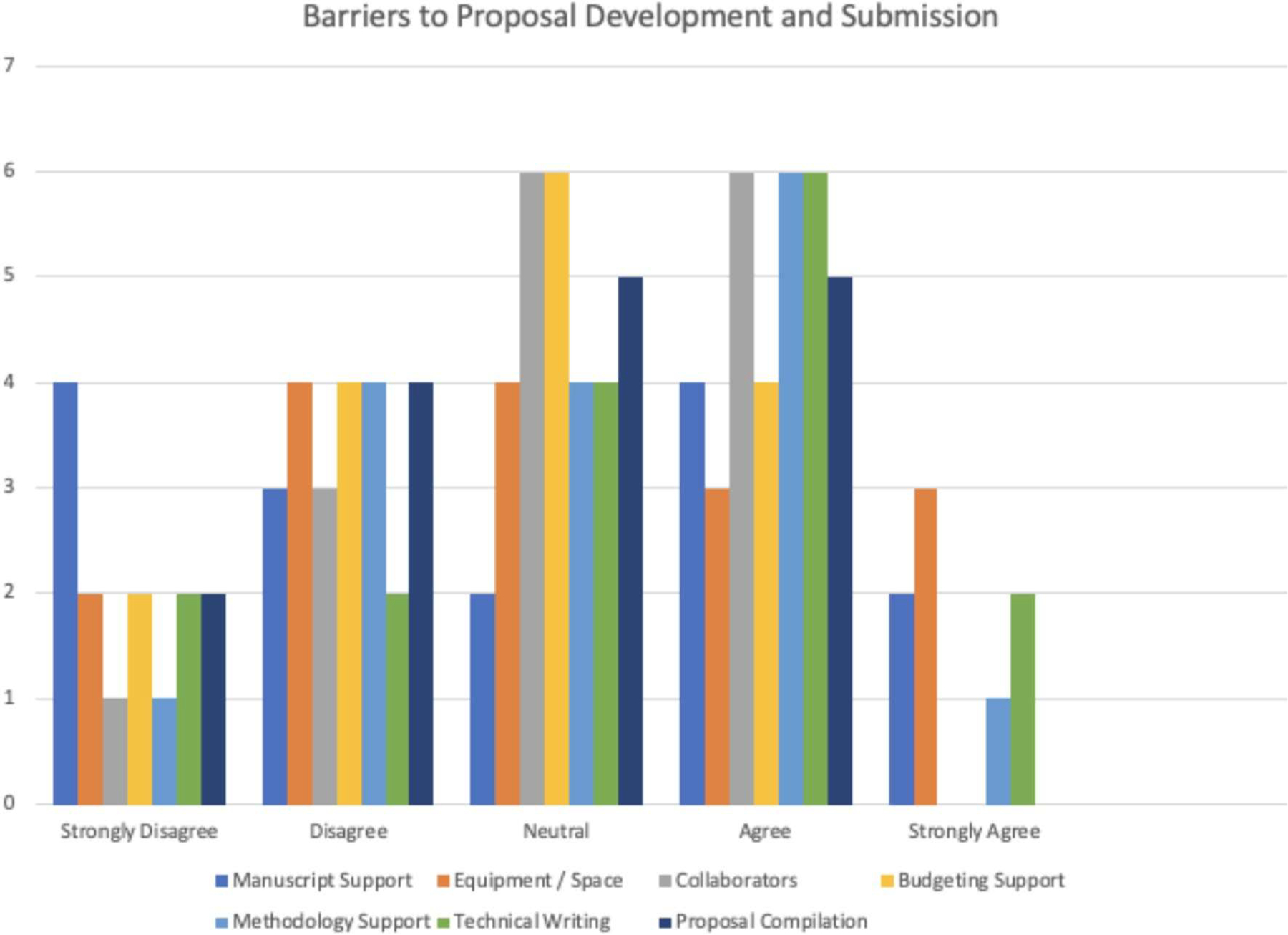
MUST researcher identified barriers to proposal development and submission

MUST researchers were also asked to rate the common barriers to success in proposal development and submission (Fig. 2).

We received 16 responses to the multiple choice option list of commonly cited barriers. Notably, the data show that manuscript support was not cited as a barrier by most respondents. However, many respondents agreed that proposal compilation, technical writing, and methodology support were needed to bolster stronger manuscript quality. This is interpreted as a proposal development barrier in that having relevant publications is a critical component of a competitive research proposal.

A representative sample of roles within MUST Research Administrators completing the survey included those in pre-award, post-award, finance, and compliance. The 10 administrators provided details regarding recent proposal submissions in which they participated. Table 2 summarizes their responses.

Additionally, respondents were asked to provide information regarding their most common Pre- and Post-Award responsibilities, which included funding opportunity identification; interpretation of sponsor guidelines; budget creation; gathering needed letters of support; award setup; budget monitoring, projection, and reporting; tracking progress performance; transaction approvals; project closeout; and mentoring of junior staff.

Both groups also reported on the tools, resources, and support that would be most beneficial to their research or administrative roles. The administrators’ free-form responses focused on sponsor-specific guidance; writing; more efficient activities/processes; automated systems; mentorship; data analysis; general research administration training; and additional staffing. Researchers were asked to rank pre-determined support categories.

**Fig. 3:**
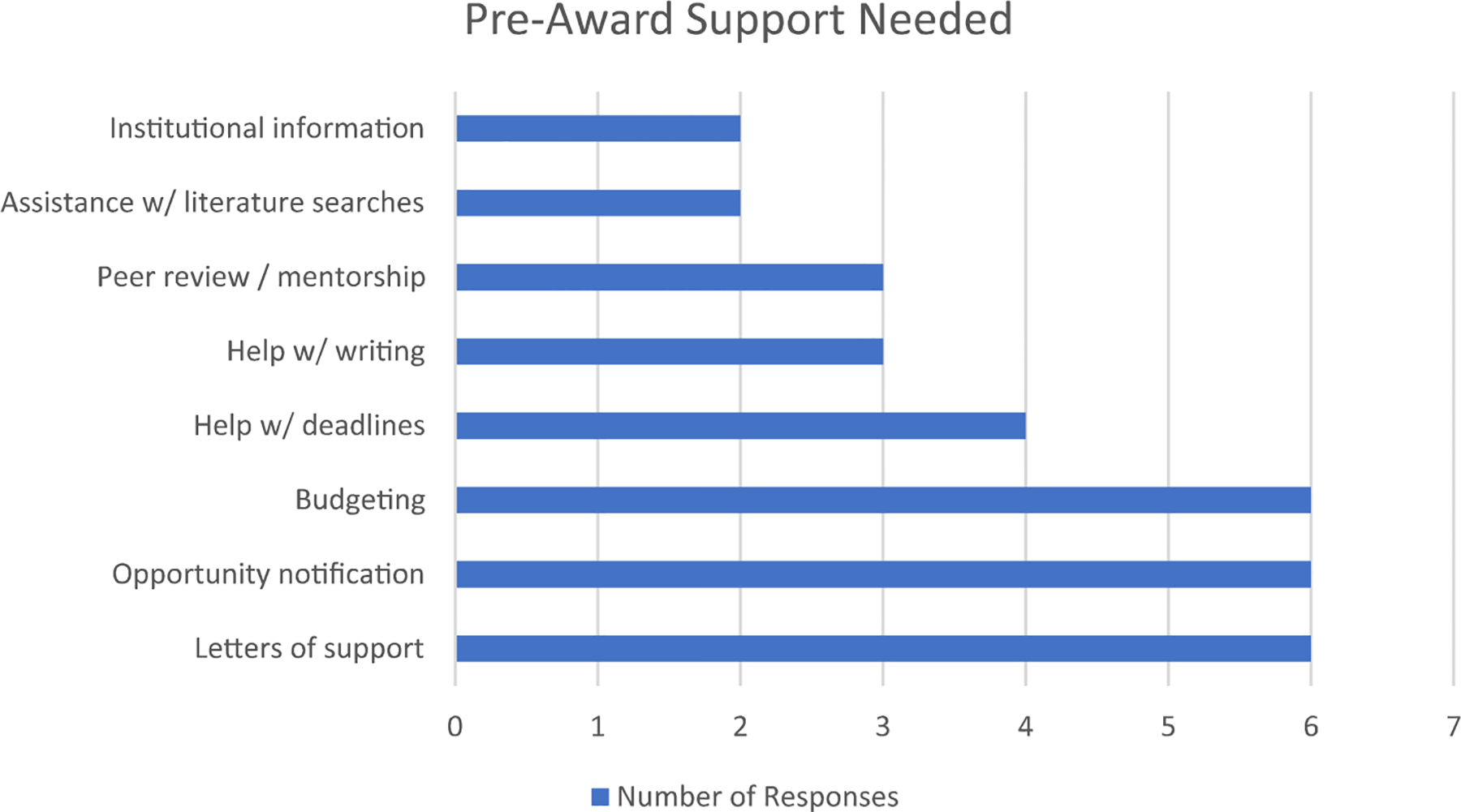
MUST researcher responses to question regarding pre-award support needed

As shown in Fig. 3, a majority of respondents cited letters of support, opportunity notification, and budgeting as areas where pre-award support was needed. Moderately, some investigators mentioned that they needed help with managing deadlines. A smaller number of researchers mentioned they needed support with institutional information, literature searches, peer review, and/or mentorship.

**Fig. 4:**
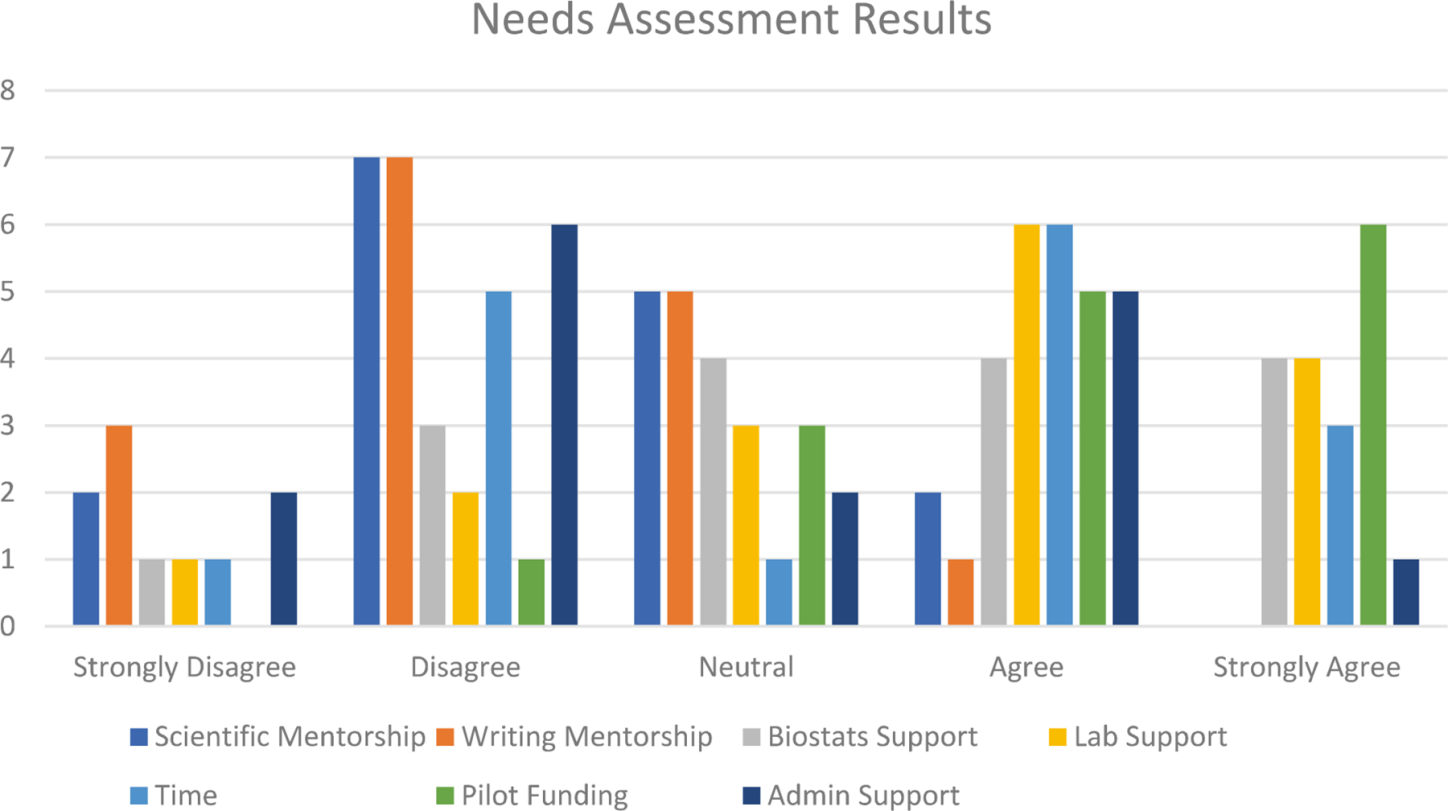
MUST researcher response regarding overall support needed to be successful in conducting research and securing research funding

Figure 4 shows that the most commonly identified needs were pilot funding, laboratory plus biostatistics support, and time management. The data show that investigators reported less need for writing and scientific mentorship plus administrator support.

### 3.3 Training, implementation, and practice

Using a mixed approach of in-person and virtual meetings, pre-existing and new seminars and workshops, along with ongoing mentorship practices, the G 11 team made significant advances in the standardization of practices, creation of usable forms and tools, and preparing the MGO to disseminate and translate their gained experiences to MUST colleagues via a “train the trainer” model.

#### 3.1.1 Training

Throughout the program, the MUST and UVA teams met on a twice monthly basis using a virtual audio/video platform to address and tackle issues specifically raised in the needs assessment survey. Together, the team created pre-award standard operating procedures, a pre-submission checklist, a proposal submission routing form, and a pre-award activity flow chart for use by administrators and researchers to better understand and streamline the pre-award processes. These documents are available on the MUST Grants Office website. The UVA team also shared template documents for standard agreement language, including material transfer and data transfer/use, to inform the creation of similar templates for agreements originating from MUST.

A major focus in Years 2 and 3 of the training program was the creation of a year-long monthly virtual presentation series on myriad topics related to international collaborative research. The sessions were led by both UVA and MUST team members, along with members of the Training Advisory Committee (Table 3). The widespread use of a virtual audio/visual platform as a connection tool due to the COVID- 19 pandemic allowed these presentations to be opened not only to MUST faculty and staff, but also to regional peer institutions in Uganda. The seminar series supported the MGO team to expand the office’s visibility to researchers and establish itself as a regional leader in the field of research administration.

In response to the survey findings, the MGO team also elected to pursue additional training for research administration fundamentals and strengthen essential pre- and post-award knowledge through the Society of Research Administrators International (SRAI), an online professional credentialing course provider. These courses provided the MUST team with replicable training materials and knowledge for dissemination to other research administration colleagues and mentees. Over the course of the program, three MUST team members completed the delivery of eight courses on subjects ranging from proposal development and pre-award management to compliance, award negotiation, and clinical research management.

#### 3.3.2 Implementation—dissemination and outreach

The project team was successful in its training program by developing, implementing, and disseminating various co-created research administration forms, processes and resources. This included providing wide and sustainable access to checklists and other relevant tools via the MUST website, as well as webinars, ad hoc one-on-one engagement, and ongoing group presentations. The most significant and permanent of these was the updated MGO website. The site has been transformed into a valuable resource for MUST researchers and administrators, housing the tools, resources, and presentations created throughout the program. The team worked in partnership with the MUST Information Technology and Web Design offices to create more visibility, intuitive formatting and organization, and useful content to support ongoing and future research activities. The implementation of these resources has equipped the MGO to provide better and more accessible services to the research community which in turn has resulted in increased researcher productivity as demonstrated by the increase in proposals and awards. This success was recognized by the Uganda Office of the Auditor General, National Office for Public Institutions when MUST was named as the premier centralized grants management office in Uganda and now serves as a Center of Excellence in the region.

#### 3.3.3 Practice-program review with training advisory committee

TAC members enthusiastically participated in the online presentation series and MUST team members were able to travel to regional peer institutions for training, benchmarking, and practice sharing. These visits further broadened the MGO’s presence as a regional leader in research administration and have precipitated collaborative training opportunities to further expand on the program’s “train the trainer” goal. MGO team members have given over 30 autonomously led training presentations to over five regional, peer institutions. Examples of presentations include, “The MUST story/experience setting up the grants office; challenges/successes and current situation”;“Setting up and managing a grants management office”;“Capacity building for research administrators/grants managers & accountants”;“Roles of Principal Investigators vis-a-vis roles of the Grants Office during pre-award, award, and post-award periods” among others.

#### 3.3.4 Achievements/lessons learned

As a direct result of the knowledge gained in both technical subject matter and mentorship strategies, the MGO, under the Directorate of Research and Graduate Training, is now more broadly networking within the community they serve to provide support and assistance to faculty and staff. This has allowed the MGO to successfully obtain and manage sponsored awards that support scholarly research activities and to ensure compliance with relevant policies and standards for fiscal and technical management of sponsored research projects. The office facilitates collaborative development of interdisciplinary proposals with respective departments and is the university’s central office responsible for reviewing and approving proposals prior to submission. As part of its expanded research development activities, the MGO also facilitates external communications and relationships with its sponsors, including domestic and foreign foundations, corporations, and government agencies in the pre-award phase and then throughout the life cycle of the award including in assisting with the preparation and submission of timely reports, invoices, and financial drawdowns.

In recognition of the MGO’s increased administrative capacity and expertise, in 2022, the Uganda Office of the Auditor General, National Office for Public Institutions, announced that the MUST Grants Office represented the premier centralized grants management office in Uganda. Now the MGO is serving as a Center of Excellence in the region for other institutions throughout Uganda to benchmark best practices in grants management, presenting and training in the management and implementation of both local and international projects and research protocols. With this heightened recognition, the MGO staff is now regularly asked to facilitate and perform in-person training at peer regional institutions such as a recent three-day training by MGO staff covering topics such as: “Why a centralized grants management Office: importance and benefits”; “Roles of Principal Investigators vis-a-vis roles of the Grants Office during pre-award, award, and post-award periods”; “Procurement using grant funds”; “The MUST experience in setting up the grants office; challenges/successes and current situation”; “Setting up and managing a grants management office”; “Grants award cycle management”; “Grants financial management best practices”; “Funding opportunities search and strategies”; “Donor systems and requirements”: “Grants related policies”; and “Capacity building for research administrators/grants managers & accountants”. Staff from the MUST Grants Office have made presentations at several local institutions including Gulu University, Makerere University, Busitema University, Bishop Stuart University, and Mountains of the Moon University, whose team traveled to MGO for benchmarking in December 2022.

Another important measurable program goal achieved is the addition of resources and presentations on an accessible MGO website to allow access to any interested parties who otherwise are unable to attend in-person events, thus reaching the widest audience possible.

Another direct result of the G 11 training program is the rise in successful research proposal submissions to the NIH, including prestigious K43 and D43 mentored and training support mechanisms. Table 4 below presents in more detail, the trajectory of primary grant proposals submitted, and awards received over a four-year span.

## 4 Discussion

Our experience demonstrates that it is feasible to implement a predominantly virtual international capacity building program for research administration. Despite the COVID-19 epidemic, the G 11 team delivered a successful training and knowledge transfer program that substantially accomplished its proposed goals via ongoing communication, consistent goal setting, regular check-ins, and using multiple available methods of training and outreach.

Data highlighted obstacles beyond the scope of our research administration focus, including support needed by researchers and investigators encompassing pilot funding and laboratory and biostatistical support. Several funders require investigators to have preliminary data or evidence of successful interventions or experiments on a small scale before testing at a larger scale. Many researchers lack access to the start-up funds that provide the proof of concept to scale and test with a larger sample size. Investigators noted a need to build laboratory capacity to support the clinical sciences. The current infrastructure provides access to clinics and patient population, but the laboratory capacity remains to be developed. Advanced laboratory infrastructure supports the conduct of more impactful research such as that involving longitudinal collection of samples [7]. These findings highlight the complexities of research and the need for more substantive support to encourage successful research outcomes.

Our G 11 grant supported capacity building for the central grants office at MUST and peripherally engaged the investigators at MUST to leverage their stated needs to guide the program’s direction. Our grant identified and highlighted existing strengths of the MGO, while comprehensively supporting the areas where capacity was needed the most. One of the most significant successes of the program was the national recognition of the performance of the MUST Grants Office and its elevation to a national Center of Excellence.

We can achieve sustainability in these process improvements through ongoing communication, education, and continued outreach by trained research administrators. To do this work, the entire institution, including transactional teams and University leadership, must share the vision of growth and advancement. To highlight successes, the MGO must present established and measurable institutional research data to the entire community so that all stakeholders are aware of growth and share in the achievements. This presents an opportunity to measure the impact of direct and transparent communication during the research development and proposal preparation process on research award outcomes.

To facilitate dissemination of this collective success, in April 2022, the UVA team members met with MUST leadership to discuss goals achieved and ongoing program activities and to obtain assurance of continued support during the team’s visit to MUST. The team is keenly aware that creating venues for education and outreach will be most successful with the explicit messaging and advocacy of institutional leadership.

## 5 Conclusion

This G 11 grant was a unique opportunity for a young research administration office such as the MGO to work closely with a more mature research administration office to learn best practices and develop tools and processes that will create standardization and efficiency. This program also gave the MGO team the opportunity to become recognized as proactive collaborators and knowledge-holders amongst their peers, both at MUST and throughout the region.

The G 11 program highlighted the disconnect between individual institutional policy and functional best practices which is further exacerbated by the complexity of international collaborations. The importance of flexibility and bidirectionality is critical to ensuring that stakeholders recognize policies as constructive rather than obstructive.

The COVID-19 pandemic may have prevented the team from holding the optimum frequency of in-person training, but by stepping up the virtual meetings and activities, the team was able to stay on track and make substantial progress. Despite these successes, the research team reiterates the need for in-person engagement in conjunction with a virtual approach.

## Figures and Tables

**Table 1:** Responses from needs assessment survey 1 from researchers at MUST

Number of responses = 16	Yes n (%)	No n (%)	No response n (%)
Are you Pl of an ongoing research project?	14 (88%)	1 (6%)	1 (6%)
Are you Pl of a completed research project?	11 (69%)	4 (25%)	1 (6%)
Success with an award in previous 12 months?	10 (62%)	6 (38%)	0
Do you intend to submit in next 12 months as prime Pl?	9 (56%)	6 (38%)	1 (6%)
Have you started but not submitted a proposal?	10 (63%)	5 (31%)	1 (6%)

**Table 2:** MUST Research Administrator response to questions regarding recent proposal activity

Number of responses = 10	
Proposals submitted over previous 12 months	Mean 1.4 [range 0–6]
Lead time for proposal preparation	Mean 6 weeks
Common sponsors	US National Institutes of Health, Centers for AIDS Research
	US Center for Disease Control
	US President’s Emergency Plan for AIDS Relief Royal Society

**Table 3:** Virtual training program schedule

Presenter	Topic	Date
1		23 Feb 2021
UVA + MUST	Introduction to the series	
MUST	Establishment of LMIC research office and key function	
2		30 Mar 2021
MUST + LMICTAC member	Proposal development, submission review and acceptance	
UVA	Budget development, roles, pre-award tools	
3		27 Apr 2021
LMIC TAC member + MUST	Grant Writing—forming & assigning roles within the writing team	
4		25 May 2021
LMICTAC member	Internal controls & research / donor compliance (audits, effort reporting etc.)	
UVA	Role of the central reviewer, institutional oversight	
5		22 Jun 2021
MUST	Role of Research Administrators (pre, post, fiscal, project management, central program)	
6		20 Jul 2021
MUST	Indirect cost policy	
MUST & UVA	Working with an international partner	
7		31 Aug 2021
MUST	Project close out SOP & Sourcing for Funding Opportunities	
8		22 Sep 2021
UVA + LMICTAC member	Networking & Managing Partnerships	

**Table 4:** MUST 4-year submission and award metrics highlight NIH and HIV/AIDS related

MUST sponsored research	2018–19	2019–20	2020–21	2021–22
# of NIH submissions	12	10	23	26
# HIV/AIDS related	10	4	9	7
% HIV/AIDS related	83.33%	40%	39.13%	26.92%
# ALL active awards	64	79	75	83
# HIV/AIDS related	12	15	20	25
% HIV/AIDS related	18.75%	18.99%	26.67%	30.12%

## Data Availability

The dataset generated from the needs assessment survey and feedback survey represents a sample size not large enough to share without compromising confidentiality.
